# Internal stress transfer characteristics of coal–rock medium under concentrated force based on particle flow method

**DOI:** 10.1038/s41598-024-55841-9

**Published:** 2024-03-08

**Authors:** Yongping Wu, Yepeng Tang, Panshi Xie, Bosheng Hu, Ding Lang, Hongwei Wang

**Affiliations:** 1 College of Energy Engineering, Xi’an University of Science and Technology, Xi’an, 710054 China; 2 Key Laboratory of Western Mines and Hazard Prevention, Ministry of Education of China, Xi’an, 710054 China; 3 State Key Laboratory of Coal Resources in Western China, Xi’an, 710054 China

**Keywords:** Energy science and technology, Engineering, Materials science

## Abstract

To solve the problem that the macroscopic deformation and failure of coal–rock medium under external loads are easy to be observed while the internal stress transfer mode and path are unclear. Based on the discrete element idea, the numerical models for pure coal or rock samples and coal–rock combination samples with different lithologies and combination methods under concentrated force are established by PFC2D software. Then the influence of coal or rock strength and combination methods on the internal stress transfer law and distribution evolution characteristics of coal–rock medium are discussed from the perspectives of macroscopic stress and mesoscopic force chain, respectively. The results showed that under concentrated load, the macroscopic stress transfer paths within pure coal or rock samples and coal–rock combination samples are primarily in the form of ‘point source radiation’. However, when transferring between coal–rock interfaces, there is a certain interface effect. For pure coal or rock samples, differences in lithology does not change the transfer rules and macro distribution patterns of internal stress, but it can cause changes in internal unit transfer stress value and local area transfer direction. For coal–rock combination samples, the greater the difference in lithology between the two sides of the interface, the more likely the interface effect will occur. In addition, the internal stress transfer is also influenced by the relative stratigraphic relationships of coal and rock. When the stress is transferred from a higher-strength rock to a lower-strength coal mass, the interface effect will be more significant. However, regardless of the combination pattern, the locations where significant stress surges occur are always within the higher strength rock mass near the interface. The findings are helpful to understand the mechanical properties and failure mechanism of mining coal and rock mass, and provide a theoretical basis for the study of the mining-induced mechanical behavior of the floor under the action of the coal pillar.

## Introduction

It is well known that the influence of mining activities will cause the redistribution of stress in the surrounding rock until a new equilibrium is formed, which is the root cause of mine ground pressure. How to effectively control and utilize redistributed stresses and then design and adjust coal mining activities is the essential connotation of mine pressure control^1^. Layered composite is a significant feature of coal-bearing strata and the coal–rock combination structure is widely present in all kinds of coal mine engineering sites, and is also the major component of all types of engineering enclosures. The study of the stress state and distribution evolution characteristics of surrounding rock is required to reveal the distribution, transmission and evolution law of mining stress within and between coal and rock.

In recent years, many scholars at home and abroad have carried out a lot of studies on coal–rock combination samples. Zhang^2^ and Chen^3–5^ studied the influence of coal and rock properties and combination methods on the mechanical properties and failure characteristics of coal–rock combination samples through uniaxial loading experiments. Yin et al.^6^ studied the influence of coal–rock height ratio on the strength characteristics of the coal–rock combination samples, and pointed out that the strength of the combinations decreases with the increase of coal height. Selçuk^7^ and Xia^8^ found that with the increase of the dip angle of the coal–rock interface, the strength of the combination will experience a trend of decreasing first and then increasing. Huang^9^ and Wang^10^ discussed the failure modes for coal–rock combination samples under different loading rates. Zuo^11^ and Song^12^ studied the mechanical properties and failure evolution characteristics of coal–rock combination samples under different loading (or unloading) modes through cyclic loading and loading–unloading experiments. Xie^13^, Liu^14,15^, Yin^16^ combined with the impact dynamic tests of coal–rock combination samples, and further studied the stress wave propagation mechanism and failure mechanism of coal–rock combination samples under dynamic and static loads. In addition, some scholars have also analyzed and studied the electrical signals^17^ and acoustic emissions^18–20^ and other precursory information^21–23^ before the deformation and failure of the coal or rock samples.

In summary, scholars' research shows that the current research on coal–rock combination samples mostly focuses on the discussion of macroscopic physical parameters such as strength, elastic modulus and deformation and failure laws, mainly by artificially changing the internal composition of coal–rock or by applying loads. However, there is still a lack of a clearer understanding of the specific stress state within the coal or rock, the process of formation and evolution, and in particular the characteristics of stress transfer between different media. While, the internal stress distribution state and transfer mode of coal–rock mass are the root cause of its macroscopic deformation and failure. In order to essentially reveal the deformation and failure mechanism of coal–rock medium, especially coal–rock combinations, it is necessary to clarify the internal stress transfer mode within the coal–rock combination, and master the transfer rules of stress within and between media, that is, to solve the basic scientific problem of stress transfer path in layered coal–rock mass, which is more representative of the study of the transfer effect of concentrated force. Based on this, with the advantages of numerical simulation visualization, this paper uses the numerical simulation software of particle flow code (PFC2D) to establish the numerical models of different types of pure coal or rock samples and coal–rock combination samples, respectively. From the macro and micro perspectives, the formation and evolution of internal stress and force chain under the action of concentrated force are compared and analyzed, and the cross-layer transfer characteristics of internal stress of coal and rock are revealed. The results of this paper will help to deepen the understanding of the internal mechanical properties and mechanism of coal or rock. Enrich the research method of the scientific problem of the internal stress transmission path of mining coal or rock mass. It can provide a theoretical basis for the mechanical analysis and stability control of the roof and floor of the working face under the action of coal (or rock) pillars in stratified mining or closed distance seam group mining.

## Model

### Particle flow code (PFC) and stress transfer

Particle flow code (PFC) numerical simulation software is a discrete element numerical simulation software developed by Itasca, which can effectively simulate discontinuous phenomena such as cracking and separation of media. It has been widely used in the field of rock mechanics to study the mechanical properties and behavior of media from the perspective of microstructure. The simulation principle can be summarized as follows: the medium consists of the basic unit particles (balls) and their interactions resulting in contact (contacts). Different types of medium bodies are simulated by setting up particles and contacts with different properties. The macroscopic mechanical properties of the media, such as the constitutive are determined by the geometric and mechanical properties of the particles and contacts. Sun^24–26^, Bouchaud^27^, Goldenberg^28^ and others in the study of granular material mechanics pointed out that the granular system has a multi-scale structure, that is, in addition to the microscopic single particle scale and the macroscopic system scale, the meso-scale force chain is the path of particle contact stress transmission. The study concluded that the particles squeeze each other to form a contact network (force chain), and the contact grid is the material basis for supporting the external load and is the main path of stress transfer. Based on this, the stress transfer characteristics of the particle system have been studied extensively. In order to explore the stress transfer rules and transmission paths of internal stress in coal and rock mass, based on this idea, this paper assumes and simplifies the internal hypothesis of coal and rock mass into particle aggregates formed by different strength cementation, and tries to analyze the transfer laws of loads inside coal and rock medium from the perspective of meso-scale force chain. 

### Preparation of coal–rock combination samples and treatment of interface parameters

Although coal–rock combination widely exists at many engineering sites in coal mining, it is difficult to directly obtain the combination samples that meet the requirements of laboratory test from the site due to the influence of coal–rock strength differences, size effects, etc. At present, most of the samples used in the study of coal–rock combination are artificial assemblages made by bonding with a cementing agent. Although this kind of samples performs well in studying the macroscopic deformation and strength characteristics of the combination, However, when studying the stress evolution at the interface, as in this paper, the artificial cementing treatment is difficult to reflect the real stress and deformation characteristics of coal–rock near the interface, and may even have an unpredictable effect on the experiment. In practice, coal and rock seams are not simply superimposed on each other, but there is often a certain transition layer (such as false roof). Some scholars also pointed out that the actual coal–rock interface is inhomogeneous and contains a large number of complex microstructures in the study of coal–rock combination samples^29,30^. In this paper, a coal–rock-like combination with a certain transitional structural surface made by pouring and processing is used as the experimental object for study. As the process of specimen preparation is not the focus of this paper, we do not go into details here, but references^31,33^ and other relevant literature studies for details. The final study determined that coal (M), rock 1# (Y1) and rock 2# (Y2) samples were made in the proportions of 1:0.45:0.8:0.45, 1:0:1.2:0.35 and 1:0.3:1.6:0.43 (cement:gypsum:river sand:water) respectively, and then the samples were poured in layers to make M_Y1 and M_Y2 combination samples on this basis.

In addition, for the internal stress transfer of coal–rock combination samples, it is necessary to distinguish the differences in the properties of each component of coal–rock, and the influence of coal–rock interface should also be considered. Therefore, some parameterization of the coal–rock interface is required when building the numerical model. According to the existing research, it can be seen that the coal–rock interface is usually deduced by a certain theoretical derivation, or even by a simple weakening of the parameters such as stiffness, friction coefficient and cohesive force, or even by ignoring the interface thickness, just for experimental experience. Some other studies assume that it has a certain thickness, but when the mechanical parameters are assigned to the area, they are mostly given a single value. Although it can reflect the interface properties to a certain extent, it will inevitably form a new contact interface with the contact coal or rock mass at the edge of the assumed interface area. 

To address this problem, analogy to the similar properties of the false roof between the coal seam and the roof, the weak interlayer of the layered rock mass and other similar coal–rock combination structure bodies, this paper proposes the transition interface hypothesis, which assumes that there exists a certain thickness of coal–rock interface and that it is a progressive transitional structural surface. At the same time, it should be emphasized that this hypothesis is not based on the strength properties of the interface, which should not be judged by the strength properties of the interface, but rather is a simplified hypothesis of the distribution attribute of mineral particles in the interface. The interface parameters cover various physical and mechanical parameters of the interface components. Specifically, in the case of coal to rock along the thickness direction within the interface, it is assumed that the correspondence between the property parameters at each location (*P*_*x*_) and their relative interface starting position (*x*), interface thickness (*h*), and the coal (*P*_*r*_) and rock (*P*_*c*_) property parameters on either side can be characterized broadly by the function relationship described in Eq. (1):

1 $$ P_{x} = P_{c} + \left( \frac{x}{h} \right)(P_{r} - P_{c} ) $$

**Figure 1:**
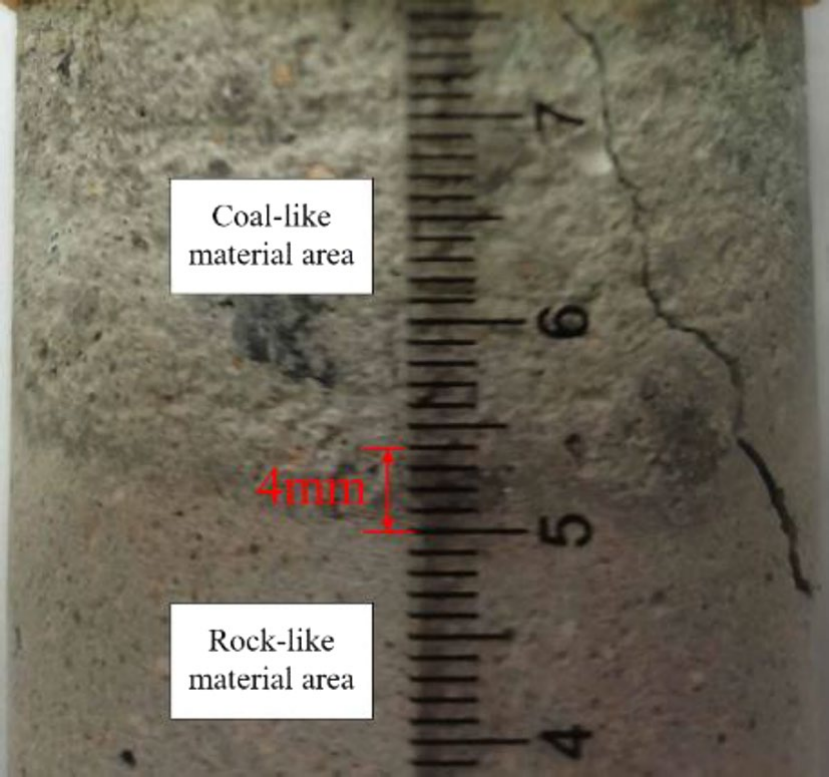
Local enlargement of the coal–rock partition interface.

This is also similar to the interface of the pour-formed coal–rock combination samples, which is measured to be 4 mm thick (Fig. 1), so the numerical model in this paper takes the interface thickness to be 4 mm and transitions some of the particles and contact parameters within the interface range according to Eq. (1).

### Calibration of microscopic parameters of coal and rock

**Figure 2:**
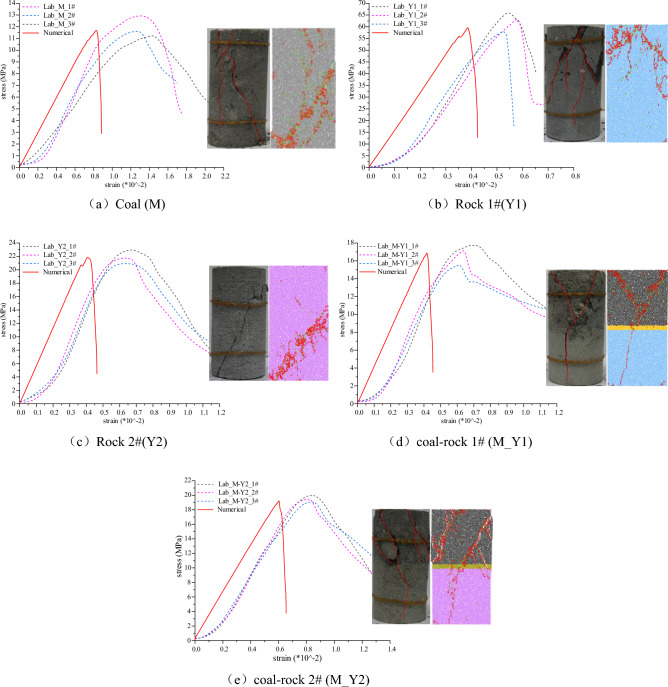
Parameter calibration results for pure coal or rock samples and coal–rock combination samples.

Usually, the parameters to be assigned to the PFC numerical model cannot be measured directly by conventional rock mechanics experiments, but have to be obtained by parameter calibration according to certain rules. As mentioned above, the influence of the interface needs to be considered for the coal–rock combination sample, so the combination model is set to a certain interface thickness (4 mm), and the interface region is parameterized for transition according to Eq. (1). Prior to the experiments, parameter calibration experiments were carried out on the models with reference to the parameter calibration methods in the literatures^34,35^, and the final calibration results of each model were obtained as shown in Fig. 2. The corresponding calibration parameter results and coal rock meso-parameters are shown in Tables 1 and 2.

**Table 1:** Coal–rock mechanical parameters and numerical model calibration results table.

Material	NO	E(Lab)/GPa	UCS(Lab)/MPa	Average E(Lab)/GPa	Average UCS(Lab)/MPa	Numerical-E/GPa	Numerical-UCS/MPa
M	M-1#	1.18	11.28	1.51	11.96	1.51	11.72
	M-2#	1.52	11.65				
	M-3#	1.82	12.96				
Y1	Y1-1#	16.8	66.13	15.8	62.54	15.90	60.70
	Y1-2#	15.2	63.14				
	Y1-3#	15.4	58.35				
Y2	Y2-1#	6.1	23.07	5.77	21.96	5.73	21.94
	Y2-2#	5.7	21.85				
	Y2-3#	5.5	20.97				
M_Y1	M_Y1-1#	4.32	17.79	4.17	16.88	4.15	17.00
	M_Y1-2#	4.28	17.25				
	M_Y1-3#	3.92	15.59				
M_Y2	M_Y2-1#	3.45	20.03	3.42	19.52	3.46	19.61
	M_Y2-2#	3.6	19.42				
	M_Y2-3#	3.21	19.12

**Table 2:** Meso-mechanical parameters of coal and rocks.

Parameter	Coal	Rock 1(Y1)	Rock 2(Y2)	Interface
Radius/(mm)	0.25–0.5	0.25–0.5	0.25–0.5	0.25–0.5
Density/(kg m^−3^)	1350	2610	2150	Transition of parameters according to Eq. (1)
Parallel bond deformation modulus/(Gpa)	1.45	16.5	4.65	
Contact modulus of the particle/(Gpa)	1.38	14.7	4.32	
Parallel bond tensile strength/(Mpa)	7.4	40.2	12.5	
Parallel bond cohesive strength/(Mpa)	10.5	48.6	19.8	
Stiffness ratio	2.20	1.21	1.74	
Coefficient of friction	0.5	0.5	0.5	
Parallel bond friction angle/(°)	26	32	28	

In addition, it should be noted that the research on rock mechanics experiment and numerical parameter calibration in this section is mainly used to verify the reliability of model parameters and interface treatment, which can provide accurate numerical parameters for the numerical model of concentrated force later. The stress state is different from the target stress studied in this paper. Therefore, the experimental results of rock mechanics will not be discussed later, focusing on the transfer characteristics within the numerical model of concentrated force action.

### Numerical model and monitoring scheme design

**Figure 3:**
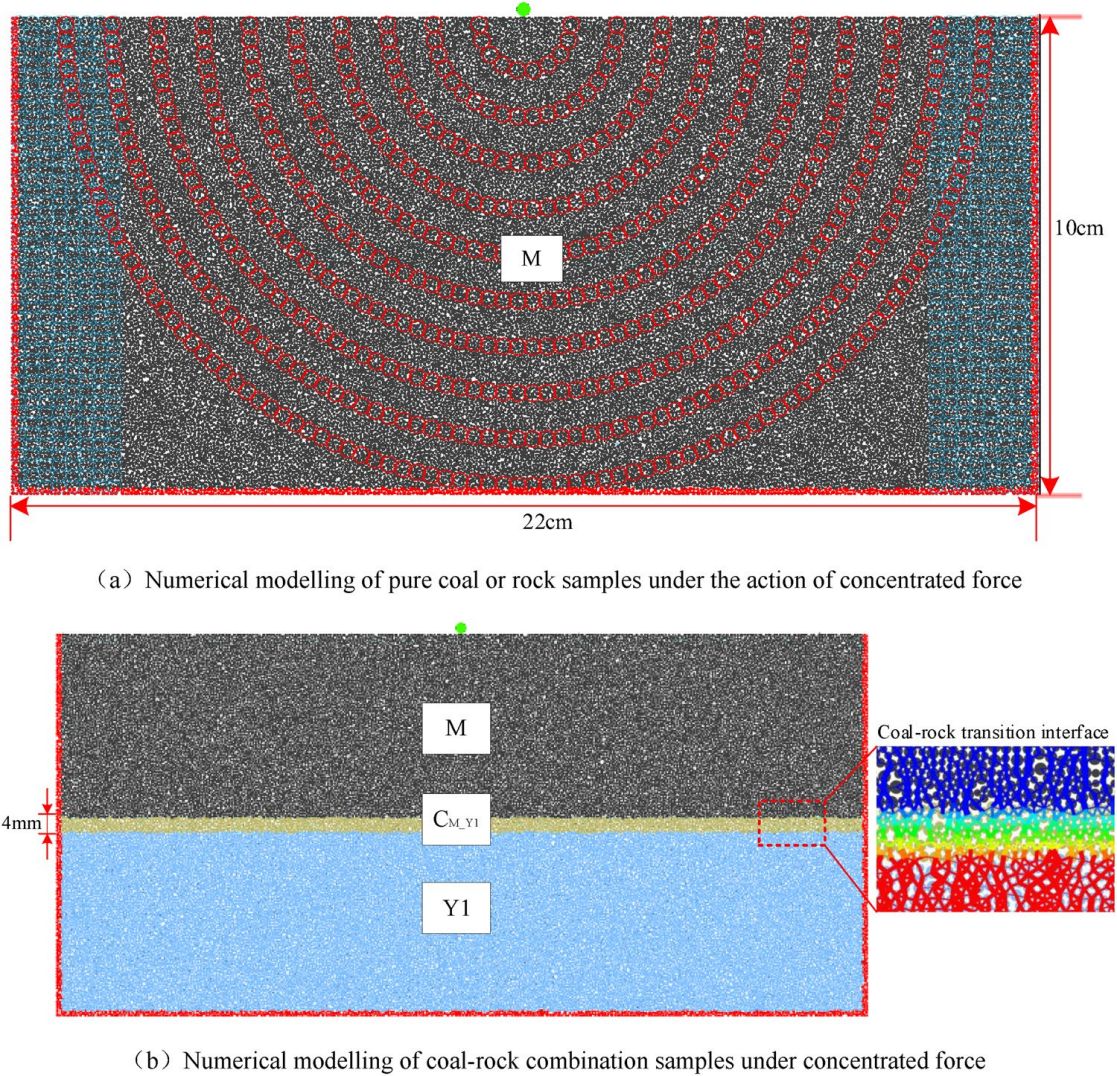
Two-dimensional particle flow numerical model under concentrated force.

Based on the characteristics of the particle flow code software and the microscopic parameters calibrated in Table 2, in order to facilitate the study of stress transfer rules, reference is made to the relevant research methods in granular material mechanics to study the transfer rules of the point load within the bulk particle system^25,28,36^. As shown in Fig. 3, two types of numerical models for pure coal or rock samples (Fig. 3a) and coal–rock combination samples (Fig. 3b) under a concentrated force are established respectively. The model size is 22 cm × 10 cm (length × height), containing 40,679 particles with a radius of 0.25–0.5 mm, of which black particles are coal (M), blue particles are rock (Y1 or Y2), and yellow particles with a thickness of 4 mm are coal–rock interface (C_M_Y1_ or C_M_Y2_). Because this paper studies the mechanical behavior of bonding materials, it is required that not only the force but also the torque can be transmitted between the particle units. Combined with the characteristics of the contact model in the PFC model^37–39^, the parallel bond model (linearpbond) was used to generate 97,375 contacts, each layer was given the corresponding lithological parameters as in Table 2, and the coal–rock interface was parametrically transitioned according to Eq. (1), and the results are shown in Fig. 3b. Both sides and the bottom of the model are fixed displacement constraints (fixed red particles). Before loading, the model is only equilibrated under its own weight. The application of the model load is achieved by setting a loading particle (green particle) at the top center, while the applied load must be relatively small to ensure that no large deformation and failure of the particle in this area causes structural reorganization. Before the formal experiment, the load P = 1 × 10^4^ N was finally determined through several sets of comparative experiments. In addition, in order to prevent the effect of dynamic loading caused by sudden loading, the fish function was applied to increase the stress gradient by 100N per 1000 steps from 0 to the preset value gradually, and after that until the model solution reached equilibrium.

In order to realize the monitoring of the internal stress of the model, 3375 measurement circles (some measures such as blue circles) are evenly arranged in each model (Fig. 3a), To facilitate monitoring of the radial stresses, 1100 circumferential measurement circles (red circles) are arranged at 1 cm intervals along the circumference, with the center of mass of the load-transmitting particle (the particle in contact with the loaded particle at the top) as the center of the circle.

## Analysis of numerical simulation results

### Internal stress transmission characteristics of a single medium under the concentrated force

The revelation of the stress transfer characteristics within the combination should be based on the premise of clarifying the influence of the coal and rock themselves on the evolution of stress transfer. Therefore, this section focuses on the numerical models of pure coal or rock samples with different strengths to analyze the stress distribution and transfer evolution characteristics within the single medium of coal or rock under the action of concentrated force.

#### Evolution characteristics of the internal force chain of a pure coal or rock sample

**Figure 4:**
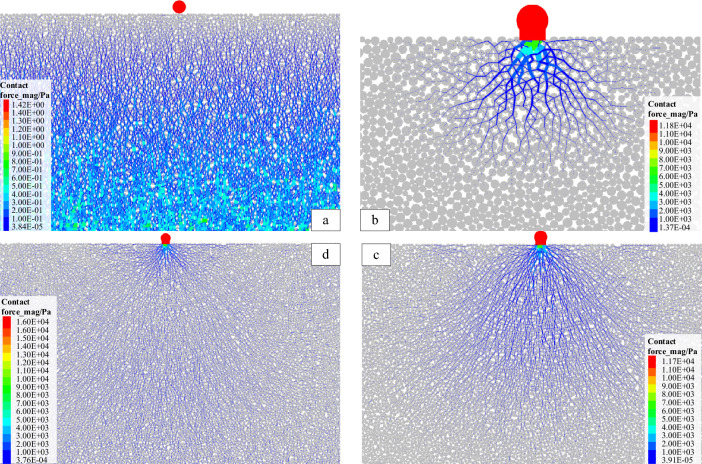
Evolution of force chain formation.

The force chain inside the PFC model is formed by the interaction between particles, which is the carrier channel for the stress transfer in the particle system. The exploration of the characteristics of the force chain can reveal the distribution, transfer and evolution of the internal stress inside the system from a microscopic perspective. The model mainly reaches equilibrium under the action of gravity before loading, and the force chain pattern of each model shows the gravity-action type force chain (Fig. 4a). When a vertical load (P = 1 × 10^4^ N) is applied to the loaded particle, the load is transmitted into the system through the contact particles, and then to the next particle through the contact of adjacent particles. In this way, the load applied at the top is transmitted to the depth of the model from top to bottom layer by layer through the interaction between the particles in the system (Fig. 4b,c). The final distribution of the force chain within the model shows that the concentrated force is transmitted in a 'point source radiation' pattern within the pure coal or rock sample, and the transfer of stress between adjacent particles is selective and directional (Fig. 4d). With the contact state of adjacent particles, the phenomenon of path diversion and size classification will occur, but the overall tendency is to transmit between particles along a quasi-linear arrangement. This is very similar to the stress transmission between granular particles, but the difference is that the particles within the coal and rock are bonded to each other, and other forms of stresses such as tensile and shear can still be transmitted between particles through bonding, so that some of the force chain patterns appear to be curved in the edge areas of the strong chains.

**Figure 5:**
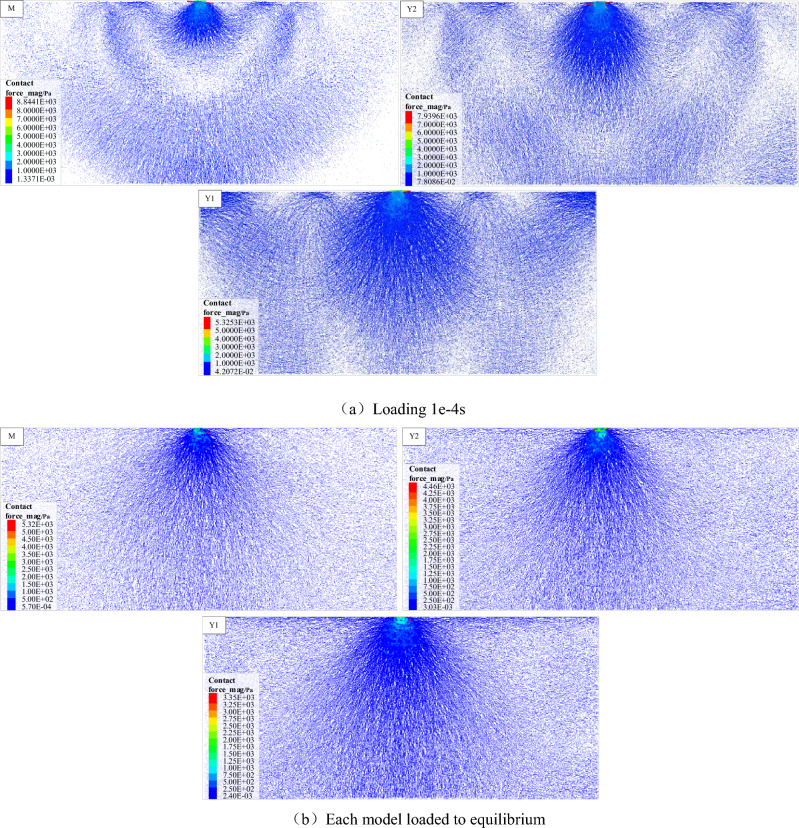
Distribution characteristics of the individual force chains of coal (M), rock 1 (Y1), and rock 2 (Y2).

As shown in Fig. 5, it can be seen from the distribution of the internal force chains within different coal or rock monoliths after loading, the difference in lithology does not change the stress transfer rules within them, but does change the value of the load-bearing and the rate of stress transfer in the internal basic units, which is reflected in a similar spatial distribution pattern of force chains but with slightly different stress values. When the loading time is the same, the higher the elastic modulus and strength of the rock mass (E_Y1_ > E_Y2_ > E_M_, σ_Y1_ > σ_Y2_ > σ_M_) the more the number of strong chains and the wider the distribution range, which can be interpreted figuratively as the faster the stresses are transmitted, and similarly distributed force chains when loaded to equilibrium. It is also due to the fact that more strong force chains bear the system stresses, that the concentration of force chains (the difference in contact stresses between strong and weak force chains within a certain area) within the relatively high strength rock mass is smaller and the maximum contact stress is lower, which may also explain to some extent the higher load-bearing capacity of this type of rock.

#### Internal stress evolution characteristics of a pure coal or rock sample

**Figure 6:**
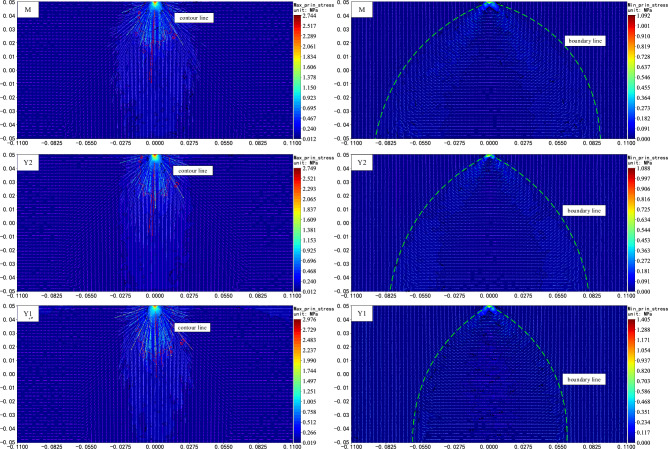
Characteristics of the principal stress distribution within the pure coal or rock sample.

Force chains belongs to the mesoscopic structure of the interaction between the internal units of coal and rock. It can be used to describe the mode of action and transfer rules of the load within the coal and rock, and to achieve a visual representation of stress transfer path. However, how to quantitatively express it is difficult, and there is a certain scale difference between it and macro stress, which are not equivalent^25^. Therefore, it is necessary to carry out further research on the distribution and evolutionary characteristics of macroscopic stresses in this structural state of the force chain. The analytical calculation of the measurement circles (blue) data was then used to plot the characteristics of the principal stress distribution for each model (Fig. 6).

As can be seen from the distribution of principal stress within the pure coal or rock sample (Fig. 6), the distribution characteristics of the maximum principal stresses within coal and rock under concentrated force are similar to the distribution state of the force chain, which is approximately radially transmitted and expanded outward with the loading point as the center of the circle, with the contours being "elliptical" in shape. The greater the strength of the rock the greater the maximum principal stress value and the greater the area of distribution of the larger stress values within the model, which manifests itself as a greater ellipse of contours (e.g., the contour line with a stress value of 0.2 MPa). At the same time, the distribution pattern of the larger value of the minimum principal stress is approximately a quadratic curve with the loading point as the vertex and the opening downward (boundary line). With the increase of rock strength, the opening of the curve gradually decreases, the distribution area of the larger value gradually shifts towards the middle, and the minimum principal stress deflection range gradually decreases.

**Figure 7:**
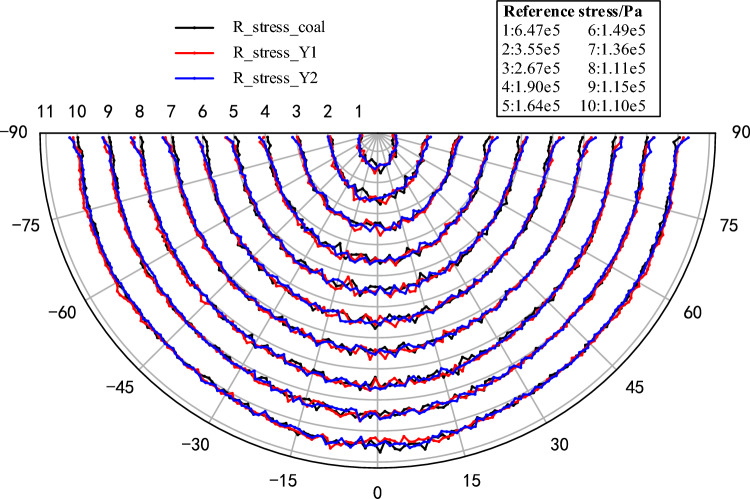
Characterization of the radial stress distribution within pure coal or rock samples.

Further, as the stress is transmitted radially within the pure coal or rock sample with the force chain grid, it will be more representative to analyze the radial stress transmission characteristics. Therefore, a polar coordinate system with the load-transmitting particle as the origin is established. By calculating the radial stress of the circumferential measurement circles at different radii, the radial stress distribution inside the model can be analyzed and plotted as shown in Fig. 7. In order to facilitate the comparison, the stress values of each layer are normalized (the values are transformed between [− 1, 1]). The 'Reference stress' in Fig. 7 refers to the average of the measured values at each radius (the gray radius line serves as a reference line, with increasing radius as positive and decreasing radius as negative; if the measured value is greater than the reference value, it is outside the radius range, and vice versa). At the same time, given that the loading of the model is achieved by loading the particle at the top, which is similar to the Flamant solution of the concentrated force on the half-plane in elastic mechanics. Therefore, in order to quantitatively characterize the transfer characteristics of the concentrated force in the pure coal or rock samples, the principal and radial stress values of each model are extracted and calculated in this paper to use the Flamant solution as a reference for comparative studies, where the radial stress at any radius can be expressed by Eq. (2)^40^:

2 $$ \sigma_{\rho } = - \frac{2F\cos \varphi }{{\pi \rho }} $$

where *ρ* is the radius, *σ*_*ρ*_ is the radial stress at the radius *ρ*, *F* is the load, and *φ* is the angle between the radial and vertical directions.

**Figure 8:**
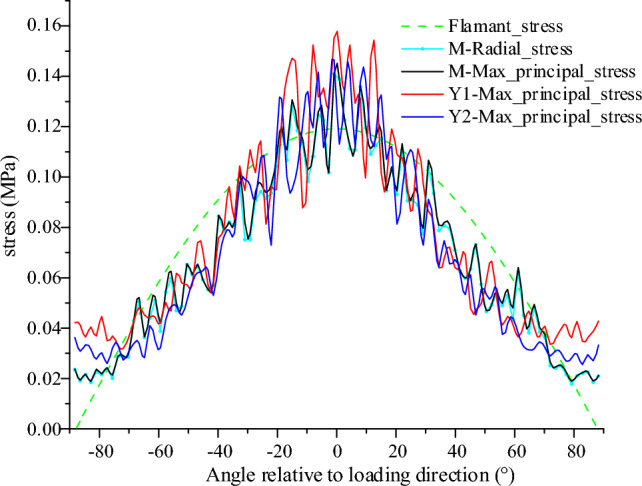
Simulated versus theoretical values at 5 cm radius.

From the radial stress distribution in the model (Fig. 7), it can be seen that the transfer of concentrated force within the pure coal or rock samples exhibits obvious single peak characteristics, that is, within the same radius, the closer to the loading direction, the greater the radial stress value, and gradually decreases to both sides, and the larger stress is mainly distributed between ± 45°. Along the radius increasing direction, the relative peak height of each layer decreases gradually, and the range of larger stresses gradually decreases. To facilitate analysis and comparison, the radial stress, principal stress value and corresponding Flamant solution of each model at a radius of 5 cm are plotted (Fig. 8). As can be seen with coal (M), although the stress value curve of local radial stress is slightly lower than the principal stress value, the difference is not significant, and the two tend to coincide, which further proves that the concentrated force mentioned above is mainly transmitted radially within the coal or rock. By comparing the principal stress and Flamant solution curves for each model, it shows that although the simulated value fluctuates greatly, it is mainly due to the limited number of particles and contacts within the measurement region and the attribution of contacts at the boundary of the measurement circles, which is a normal experimental error. It can be considered that the distribution pattern of the simulated curve is basically consistent with the theoretical value.

Based on the distribution and transfer characteristics of internal force chain, radial stress and principal stress of pure coal or rock samples with different strength, it can be seen that under the action of concentrated force, the meso-scale force chain morphology and macroscopic stress transfer paths inside pure coal or rock samples have strong consistency, it takes the loading point is a 'point source radiation' distribution along the radial path towards the loading side. For pure coal or rock samples, the difference in lithology does not change the transfer rules and macroscopic distribution patterns of internal stress within coal and rock, but it does cause changes in the stress transmission value of internal units and the direction of stress transmission in local areas, which will inevitably cause differences in macroscopic deformation, bearing and stress transmission characteristics of different lithologies.

### Characteristics of stress transmission within coal–rock composite medium under concentrated force

**Figure 9:**
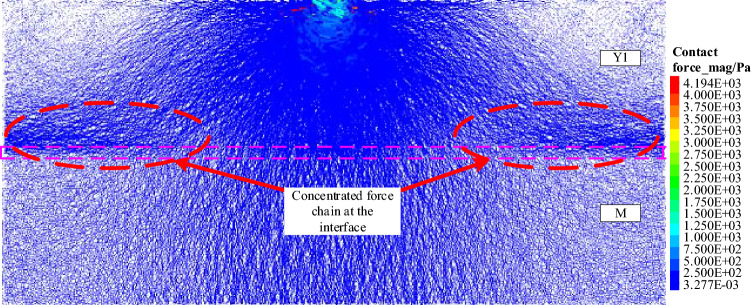
Characteristics of the internal force chain distribution in the coal-rock combination samples under concentrated force.

In the simulation experiments on the stress transmission characteristics of coal–rock combination samples, it was found that although the majority of force chains within the combination are similar to those of the pure coal or rock samples in a radial pattern. However, there was an obvious concentration of strong chains in the region of the rock near the coal–rock interface, which is the main difference between the coal–rock combination samples and the pure coal or rock samples (Fig. 9). Therefore, the following study on the stress transmission characteristics of coal–rock combination samples will focus on the stress distribution and transmission pattern near the coal–rock interface.

#### Evolution characteristics of internal force chain of the coal–rock combination samples

**Figure 10:**
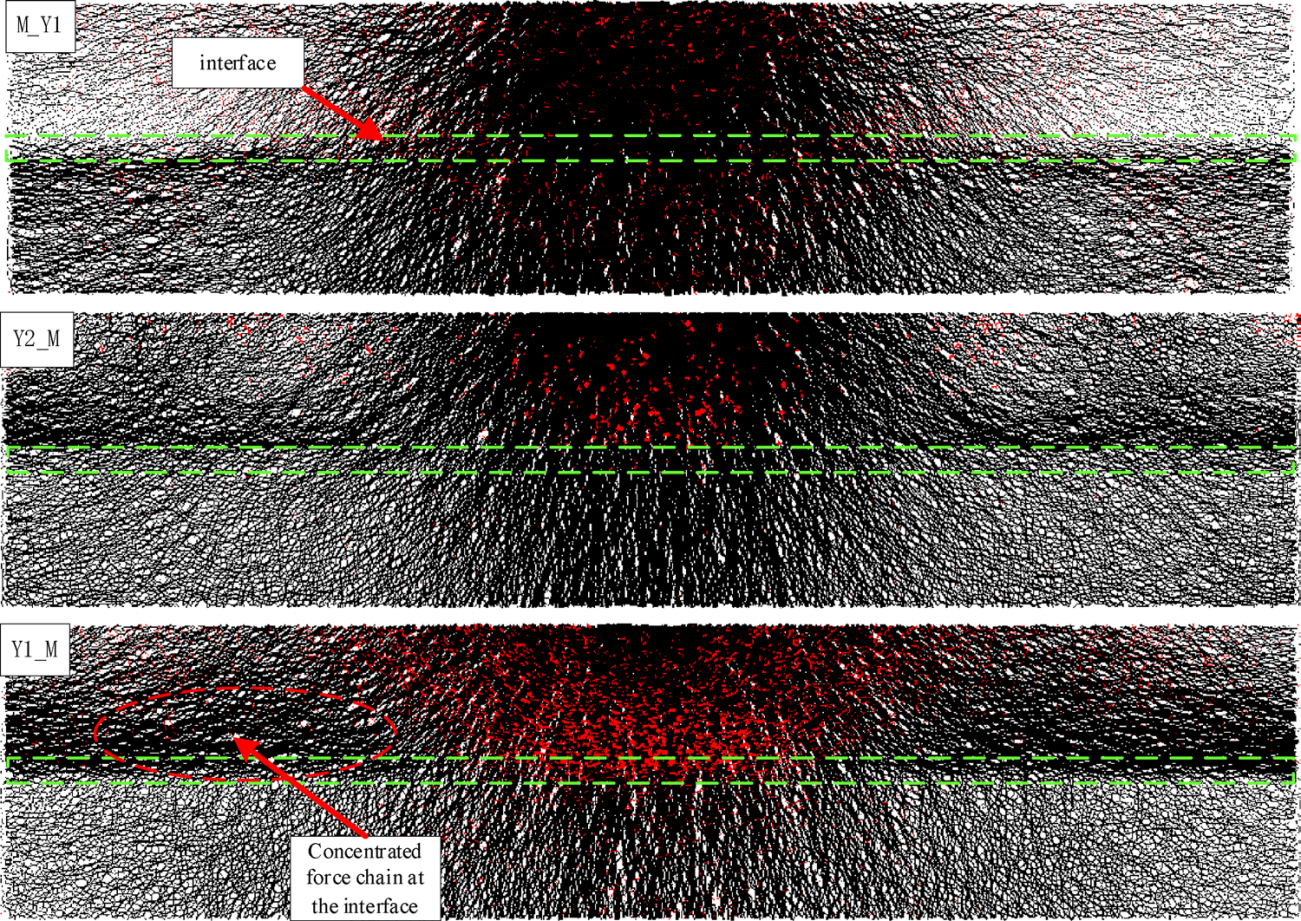
Distribution characteristics of force chain at the coal–rock interface (pressure force chains in black, tension force chains in red).

It can be seen from the force chain characteristics of each model (Fig. 10): as the stresses are transmitted hierarchically within the coal or rock through particle contact with different stress magnitudes and directions, and subsequently form a grid of force chains with different directions and differential strengths. Therefore, in a certain side of the rock mass of the combination, as the particles and contact properties are consistent in the local range, the degree of difficulty of stress transfer between the particles is similar, mainly to form a stable linear force chain, so the force chain in this region is quasi-linear and radial distribution. When close to the interface, due to the change in lithology, the properties of the particles and inter-particle contact will change, resulting in local changes in their load-bearing capacity and stress environment, which in turn leads to changes in the stress transfer rules and paths between the particles, manifested as the adjustments in the strength and direction of the force chain, and the transformation of the original linear force chain into a complex non-linear force chain grid. As shown in Fig. 10, the force chain will be bent near the interface, and the strength grading will form a force chain concentration along the layer. In addition, the change in lithology even causes a change in the type of force chains on both sides of the interface. It can be seen that although the force chain types in the rock area of Y1_M and Y2_M are dominated by pressure-type force chains, there are still more tension-type force chains. While the number of tension-type force chains decreases significantly when crossing the interface to reach the coal mass.

By comparing the force chain distribution characteristics of the different combination models, it can be seen that the force chain properties and distribution patterns within the coal–rock combination samples are influenced by the lithology in addition to the relative stratigraphic relationships between the coal and rock. As shown by the force chains near the Y1_M and M_Y1 interfaces, although the two lithologies within the combination are identical, the force chains concentration in the rock mass near the Y1_M interface are more significant, and the type of force chains on both sides of the interface changes greatly. In addition, a comparison of the force chain diagrams for the three combinations also shows that the concentration of force chains is more likely to form when the stress is transmitted from the higher strength rock into the lower strength coal mass, and the greater the difference in lithology between the two sides of the interface, the greater the concentration of the force chain in the rock area with higher strength near the interface.

#### Internal stress evolution characteristics of different coal–rock combination samples

The distribution and evolution of the mesoscopic force chain structure is the source of the realization of the internal stress transfer. Differentiated force chains near the interface of the combination are also bound to cause different characteristics of the macroscopic stresses in this region to some extent.

**Figure 11:**
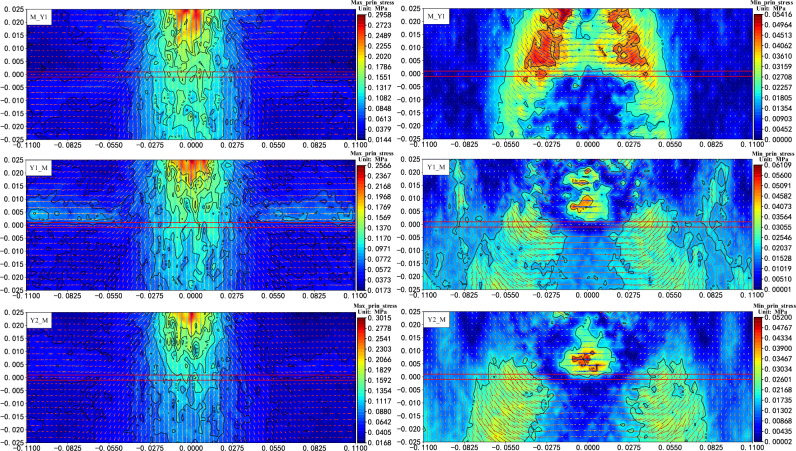
Characteristics of the principal stress distribution near the interface of the coal–rock combination samples (vertical axis − 0.002 to 0.002 is the coal–rock interface).

**Figure 12:**
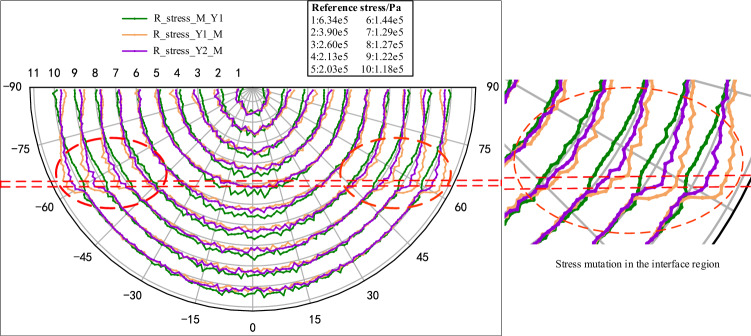
Characteristics of the radial stress distribution within the coal–rock combination samples for different combinations.

It can be seen from the principal stress distribution near the coal–rock interface that the characteristics of the maximum principal stress distribution at the coal–rock interface are in strong agreement with the force chain distribution pattern in the region (Fig. 11), that is, when the load is transmitted into the rock mass, it is first transmitted in a radial path to the depth, and when it reaches the vicinity of the coal–rock interface, the transfer stress value and direction change appear in each model, followed by a stress transmission along the level and a sudden increase or decrease in the stress value in the local area on both sides of the interface (hereinafter referred to as the "interface effect"). Similarly, under the influence of the interface effect, it can be seen that the transfer stress value and distribution pattern of the minimum principal stress on both sides of the interface have changed greatly. And, as shown in Fig. 12, this sudden change in stress near the interface caused by a change in lithology is also visible in the radial stresses within the model.

Combining the above studies, for the pure coal or rock samples, the Flamant solution in elastic mechanics can be used to approximately describe the distribution and transmission characteristics of the concentrated force within the monomer. In the coal–rock combination samples, due to the difference in lithology between the two sides of the interface and the specificity of the interface properties, the principal stresses and radial stresses in the vicinity of the interface change considerably in each model. The characteristics of Flamant solution based on radial transmission seem not to be applicable to coal–rock combination samples. However, given that there is no suitable theory to characterize the cross-layer transfer and distribution of stresses, and there is still a strong similarity to the Flamant solution in terms of macroscopic stress distribution patterns. Therefore, in order to achieve a quantitative description of stress transfer at the interface within the combined body, the stress distribution characteristics of the Flamant solution are used as a reference base for comparative studies, and then introduces the parameters of stress increment and direction change coefficient to further characterize it. The stress increment means the difference between the maximum principal stress value at any position of the combined model and the radial stress value corresponding to the Flamant solution at this point. The direction change coefficient is the difference between the directional angle of the maximum principal stress at any position in the combined model and the directional angle (radial direction) of the Flamant solution at that point divided by 90° (the range of values is [− 1, 1], with the point corresponding to the radial direction as the reference, and the direction away from the load on both sides is positive, and the opposite is negative). Based on this, the maximum principal stress increment and direction change coefficient near the interface of the combined model can be plotted as follows.

**Figure 13:**
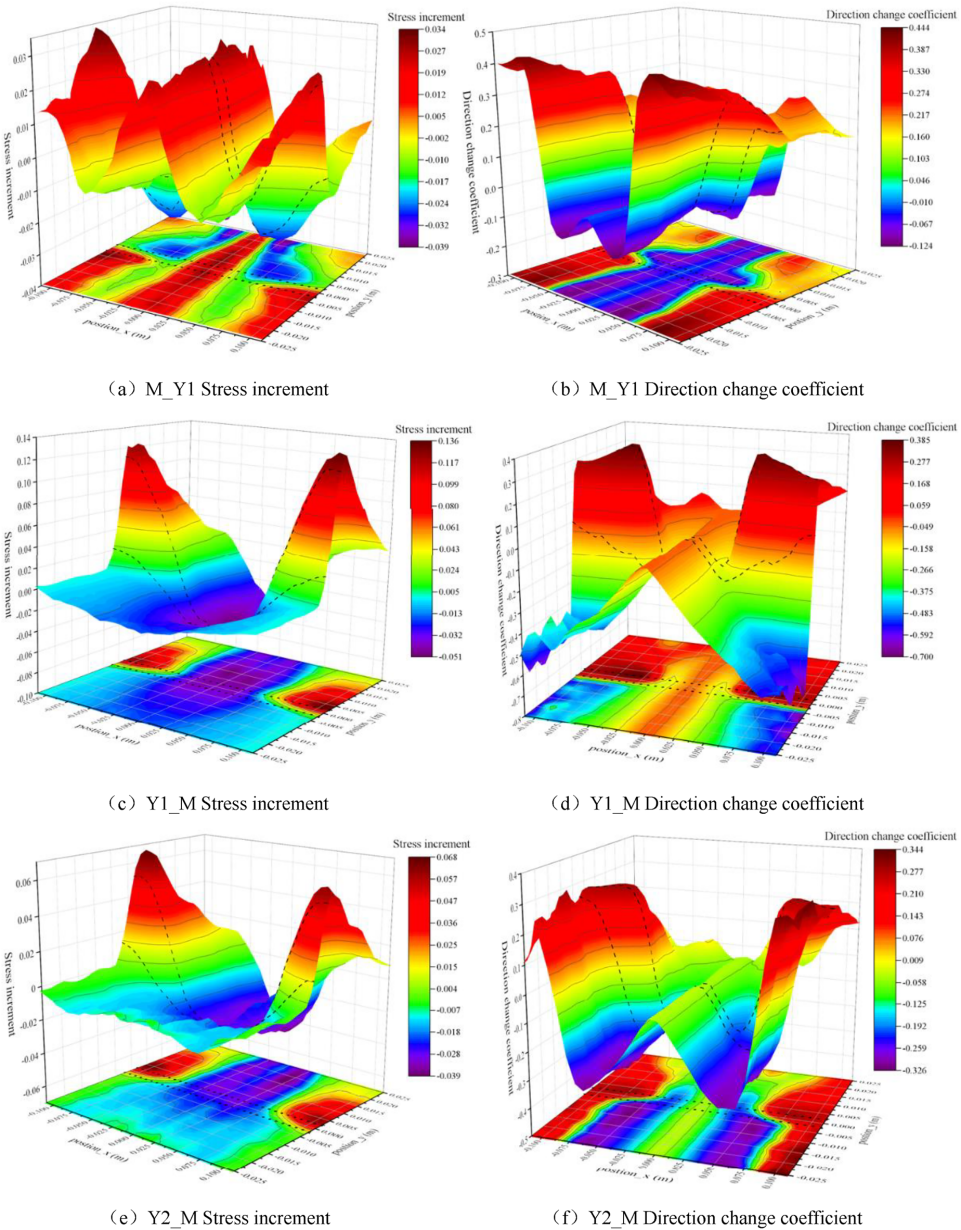
Increment of principal stress value and direction change coefficient near the interface of combined model (the black dashed line shows the position of the interface boundary).

As can be seen from the characteristics of the principal stress distribution near the interface of the combination model (Fig. 13), there is a certain degree of "interface effect" within the combination, regardless of the way in which the coal and rock are combined, and it is manifested in both the value and direction of the transfer stress, but the significance varies slightly with the combination methods. Specifically, when the relative stratification of coal and rock is the same (Y1_M, Y2_M), the greater the difference in lithology between the two sides, the greater the gradient and maximum value of the graphical change in the stress increment and direction change coefficient, and the more significant the "interface effect". If the lithology is the same on both sides of the interface (Y1_M, M_Y1), sudden changes in stress and directional adjustments near the interface are more likely to occur when stress is transmitted from the rock into the coal mass. At the same time, a comparison of the characteristics of the principal stress distribution under the three combinations shows (Fig. 13) that whether the stress is transmitted from the rock to the coal mass or from the coal to the rock, the locations where stress surges occur and are transmitted along the interface are always found within the higher strength rock mass near the interface.

In summary, the macroscopic stress transfer characteristics within the coal–rock combination samples under the concentrated force are similar to those of the pure coal or rock samples, both of which are dominated by point source radiation, but it shows a strong interface effect during the transfer between the coal and rock interface, which is manifested in the form of non-linear force chain concentration and sudden stress change along the layer direction near the interface. At the same time, lithological differences and relative stratigraphic relationships within the combination can have varying degrees of influence on the internal stress transfer. However, regardless of the combination methods, there is always a force chain concentration and a sudden increase of stress along the layer in the higher strength rock near the interface, and it is more significant when the stress is transmitted from the higher strength rock into the lower strength coal mass. This is because for one side of the rock, as the lithology of the area is uniform and approximately isotropic, the movement and stress transmission capacity of the particles and contacts in all directions are similar, so the area is similar to the monolithic model, and the stress transfer follows the monolithic transfer rule in a quasi-linear pattern in the radial direction. When close to the interface, as the lithology changes, the bearing capacity and deformation characteristics (elastic modulus, Poisson's ratio, etc.) of the particle set also change. The deformation of the rock with higher elastic modulus is more restricted by its own properties, and the deformation is smaller. The load transmitted from the top is difficult to achieve effective release and transfer, so it is easy to form the stress concentration along the layer, and then the phenomenon of transfer along the layer occurs, resulting in the incoming stress on the coal mass side also changes, and this characteristic will become more prominent with the increase of the difference of lithology on both sides.

## Discussion

In this paper, the use of PFC particle sets to simulate coal and rock mass to explore internal stress transfer from a force chain perspective is a reference to the methods used in particle material mechanics to study stress transfer in granular systems. However, the difference is that although the smallest unit of the model is still the particle, there is a strong bond between the particles using the PBM model and the model base is actually a combination of voids, particles and contacts. For coal or rock, the basic composition is also consists of mineral particles, cements and voids, which is similar to the composition of the model. For this simplification, this paper considers that it is feasible to use PFC numerical software combined with particle material mechanics to analysis and explore the stress transfer characteristics inside coal and rock media.

**Figure 14:**
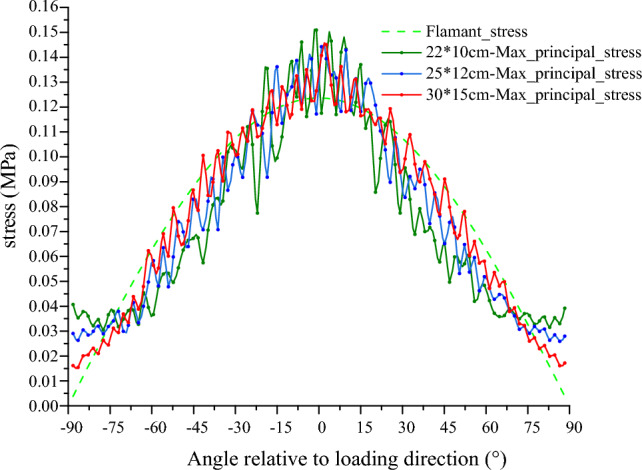
Comparison of radial stress for different model sizes (radius of 5 cm).

During the numerical experiments, it was found that the force chain pattern and radial stress distribution within the upper part of the coal or rock mass, which is in direct interaction with the loading point, exhibited a point-source radiation pattern in both the monomer and combined models. Along the circumferential direction, the stress decreases from the middle to the sides, and as mentioned earlier, this stress distribution pattern is also more consistent with the Flamant solution in elastic mechanics. However, it is worth noting that, compared to the evolution of the Flamant theoretical solution (Fig. 8), the stresses measured in the numerical experiments increase close to ± 90° (near the upper surface of the model), while the stresses in this region should remain at a reduced value or even at zero according to the Flamant solution. To address this issue, the analysis suggests that this may be related to the size effect of the numerical model. The Flamant solution is based on a semi-infinite plane and there are fixed boundaries in the lateral direction of the model (horizontal stress transfer phenomena exist at the interface and servo boundaries should not be used). To address this issue, the analysis suggests that this may be related to the size effect of the numerical model. The Flamant solution is based on the semi-infinite plane, while there are fixed boundaries in the lateral direction of the model (the interface has horizontal stress transfer phenomena, and it is not appropriate to use servo boundaries). The surface particles also have some horizontal deformation and displacement after being loaded, and the horizontal stress increases due to the influence of the lateral boundaries. In order to verify this point of view, we set up two other groups of comparison experiments in addition to the above experimental models, and at the same time increased the size of the reference group model in the horizontal and vertical directions, respectively, to establish two groups of reference models of 25 × 12 cm and 30 × 15 cm (due to the influence of computer computing power, it is difficult to run too large models). Using the same research methods as above, a comparison of radial stresses was extracted (Fig. 14). It can be seen that after increasing the size of the model, the boundary effect of the model is significantly reduced. At the same time, by comparing the radial stress curves of different lithologies in Fig. 8, it shows that the stress value in this region also has a certain relationship with lithologies. However, as the purpose of this paper is to discuss the macroscopic stress transfer characteristics within the coal or rock mass, so the influence of this region is ignored for the time being in this study on stress transfer and the radial stresses are considered to be consistent with the Flamant solution. As for the specific formation and mechanism of action of this phenomenon, it is expected to be continued in the next research.

**Figure 15:**
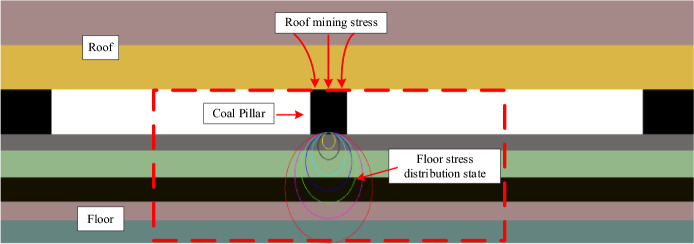
The schematic diagram of stress distribution characteristics of floor under the action of coal pillar.

Admittedly, it is rare to encounter concentrated load action in actual coal mine engineering, However, this solution makes it possible to know the rules for the transfer of stresses within the rock mass and to solve the problem of stress distribution under other forms of loading conditions by integral methods^1^. This paper is an analytical study of the theoretical model, which does not involve specific engineering applications for the time being. However, the study of the internal stress transfer model of the coal or rock medium under the action of a concentrated force will help to improve the understanding of the stress environment of the surrounding rock at the site of such projects, and subsequently improve the maintenance and construction of the relevant engineering structures. For example, as shown in Fig. 15, in the case of stratified mining or closed distance seam group mining, after the working faces on both sides of the coal pillar have been mined, the overburden mining stresses will be transmitted through the coal pillar to act on the floor. In comparison, the size of coal pillar is much smaller than that of floor. In this case, when studying the distribution and evolution characteristics of floor mining stress under the action of coal pillar, the structure can be simplified into a similar analysis model of layered coal and rock mass under the action of concentrated force. And it is our ongoing research work, more research will be carried out gradually in the later stage. In addition, at the macro scale, it is similar to the supporting effect of coal (or rock) pillars on the roof and floor in the room-and-pillar method mining, and the support of small section support such as single support and pillar in the working face or roadway to the coal gangue interbedded roof, and the loading effect of overburden stress arch footings on the surrounding rock of face, the scale difference between the loading range and the carrier can be simplified to a certain extent as the action model of concentrated force acting on coal–rock combination samples (or combined rocks).

In addition, it should be noted that the study of the transfer of stresses within the coal and rock under the action of concentrated force in this paper focuses on the coal and rock in the elastic phase, and does not involve macroscopic deformation and failure, but it can reflect the strength characteristics of the combination from the side to a certain extent. For example, the non-uniformity of the stress distribution within the combination is the root of the mechanics that determines the initial failure location of the combination.

The 'interface effect' described in this paper may be another mechanical explanation for the combination's appearance where the strength of the stronger rock near the interface decreases, while the strength of the weaker coal increases, and the strength of the combination lies between the strength of the two individual bodies within, but the specific mechanical mechanism of action is to be developed progressively in later studies.

## Conclusions

Through the numerical simulation experiment on the internal stress transmission characteristics of different pure coal or rock samples and coal–rock combination samples under concentrated force, the following conclusions can be obtained:

1. Under the action of concentrated force, the stress transfer path within the pure coal or rock samples is predominantly 'point-source radiation', with the stress contour being approximately elliptical in shape, as a result of selective transfer between internal contact units. For coal–rock combination samples, changes in lithology do not cause changes in internal stress transfer rules and macroscopic distribution patterns, but do cause changes in internal unit transfer stress values and local area transfer directions, which in turn induces differences in the macroscopic deformation, bearing and transfer characteristics of different lithologies.
2. Under the action of concentrated force, the internal stress transfer in the coal–rock combination samples shows a strong 'interface effect'. The macroscopic manifestation is the transfer of stress along the layer and the formation of stress mutations on both sides of the interface, and microscopically manifested by the reduction of the quasi-straightness of the force chain and the grading of the strength difference forming the concentration of the force chain along the layer, and even causing the change of the stress transfer properties of the force chain on both sides of the interface. It is the inevitable result of the cross-layer transfer of stress from one rock mass to another, and this phenomenon will become more pronounced as the difference in lithology between the two sides increases.
3. The internal stress transfer path within the coal–rock combination samples is strongly influenced by the properties of the coal–rock and the relative stratigraphic relationships. The greater the difference in lithology on both sides of the interface, the more significant the 'interface effect' of internal stress transfer will be; Concentration of force chains and sudden stress changes along the layers are more likely to occur when stresses are transferred from the higher strength rock to the lower strength coal mass; However, regardless of the method of coal–rock combination, the location where stress surges occur is always within the higher strength rock near the interface.
4. The evolution of contact force chains and principal stress distribution within the pure coal or rock samples and coal–rock combination samples under a concentrated force has a high similarity and can be used to characterize the stress transfer paths within the model. Among them, the distribution pattern and the evolution of the transfer paths within the pure coal or rock samples exhibit a gradual transition characterized by a continuous transfer. The macroscopic stress transfer within the coal–rock combination samples will undergo a continuous-discontinuous-continuous process, that is, the transfer is continuous within a single rock on one side, while a large change in stress value and transfer direction occurs near the interface, which can be regarded as a macroscopic discontinuity.

## Data Availability

The data that support the findings of this study are available from the corresponding author upon reasonable request.

## References

[1] Qian M, Shi P, Xu J (2010). Ground Pressure and Strata Control.

[2] Zhang Z, Liu J, Wang L, Yang H, Zuo J (2012). Effects of combination mode on mechanical properties and failure characteristics of the coal rock combinations. J. China Coal Soc..

[3] Chen G (2023). Determination of bursting liability of coal–rock combined body based on residual energy release rate index. Chin. J. Rock Mech. Eng..

[4] Chen G, Li Y, Li T, Zhang G (2023). Experimental study on the mechanical properties of intermittent jointed sandstone considering water-rock interaction and confining pressure effect. Bull. Eng. Geol. Environ..

[5] Chen GB, Wang EY, Wang WC, Li T, Zhang GH (2019). Experimental study on the influence of lithology and rock-coal height ratio on mechanical properties and impact effect of combined body. Energ Source Part A.

[6] Yin DW, Chen SJ, Sun XZ, Jiang N (2019). Strength characteristics of roof rock-coal composite samples with different height ratios under uniaxial loading. Arch. Min. Sci..

[7] Selçuk L, Aşma D (2019). Experimental investigation of the rock–concrete bi materials influence of inclined interface on strength and failure behavior. Int. J. Rock Mech. Min. Sci..

[8] Xia ZG (2021). Mechanical properties and damage characteristics of coal-rock combination with different dip angles. Ksce J. Civ. Eng..

[9] Huang B, Liu J (2013). The effect of loading rate on the behavior of samples composed of coal and rock. Int. J. Rock Mech. Min. Sci..

[10] Wang N, Xu YQ, Zhu DY, Wang N, Yu BF (2018). Acoustic emission and failure modes for coal-rock structure under different loading rates. Adv. Civ. Eng..

[11] Zuo J, Xie H, Meng B, Liu J (2011). Experimental research on loading-unloading behavior of coal-rock combination bodies at different stress levels. Rock Soil Mech..

[12] Song SL (2020). Study on failure modes and energy evolution of coal-rock combination under cyclic loading. Shock Vib..

[13] Xie BJ, Chen DX, Ding H, Wang GY, Yan Z (2020). Numerical simulation of split-Hopkinson pressure bar tests for the combined coal-rock by using the Holmquist–Johnson-cook model and case analysis of outburst. Adv. Civ. Eng..

[14] Liu S (2014). Nonlinear catastrophy model and chaotic dynamic mechanism of compound coal-rock unstable failure under coupled static–dynamic loading. J. China Coal Soc..

[15] Liu S, Mao D, Qi Q, Li F (2014). Under static loading stress wave propagation mechanism and energy dissipation in compound coal-rock. J. China Coal Soc..

[16] Yin G, Li X, Lu J, Li M (2017). Disaster causing mechanism of compound dynamic disaster in deep mining under static and dynamic load conditions. J. China Coal Soc..

[17] Zhao Y, Jiang Y, Zhu J, Sun G (2008). Experimental study on precursory information of deformations of coal-rock composite samples before failure. Chin. J. Rock Mech. Eng..

[18] Yang S, Wang J, Ning JG, Qiu PQ (2019). Experimental study on mechanical properties, failure behavior and energy evolution of different coal-rock combined specimens. Appl. Sci. Basel.

[19] Zhang H (2020). Numerical investigation on crack development and energy evolution of stressed coal-rock combination. Int. J. Rock Mech. Min. Sci..

[20] Zuo J, Pei J, Liu J, Peng R, Li Y (2011). Investigation on acoustic emission behavior and its time-space evolution mechanism in failure process of coal-rock combined body. Chin. J. Rock Mech. Eng..

[21] Chen L (2023). Experimental study on influence of lithology on directional propagation law of type-I cracks. J. Central South Univ..

[22] Zhang W, Guo W, Wang Z (2022). Influence of lateral pressure on mechanical behavior of different rock types under biaxial compression. J. Cent. South Univ..

[23] Zhao T (2022). Controlling roof with potential rock burst risk through different pre-crack length: Mechanism and effect research. J. Cent. South Univ..

[24] Sun Q, Jin F, Liu J, Zhang G (2012). Understanding force chains in dense granular materials. Int. J. Mod. Phys. B..

[25] Sun Q, Wang G (2009). Granular Matter Mechanical Introduction.

[26] Sun Q, Wang G, Hu K (2009). Some open problems in granular matter mechanics. Progress Nat. Sci..

[27] Bouchaud J-P, Claudin P, Levine D, Otto M (2001). Force chain splitting in granular materials: A mechanism for large-scale pseudo-elastic behaviour. Eur. Phys. J E.

[28] Goldenberg C, Goldhirsch I (2002). Force chains, microelasticity, and macroelasticity. Phys. Rev. Lett..

[29] Ju Y (2022). Transparentized solutions and interpretation for the effects of discontinuous structures and multiphysics on rock failure. J. China Coal Soc..

[30] Wang K (2023). Numerical simulation of interface mechanical effects of primary coal-rock combination. Rock Soil Mech..

[31] Huang Y, Yang S, Liu X (2014). Experimental and numerical study on the mechanical characteristics of rock-like materials. J. Exp. Mech..

[32] Yin P (2016). Experiment and Particle Flow Simulation on Mechanical Properties of Layered Composite Rock Under Uniaxial Compression.

[33] Zuo B-C, Chen C-X, Liu C-H (2004). Research on similar material experiment. Rock Soil Mech..

[34] Shi C, Yang W, Yang J, Chen X (2019). Calibration of micro-scaled mechanical parameters of granite based on a bonded-particle model with 2D particle flow code. Granul. Matter..

[35] Wu H, Dai B, Zhao G, Chen Y, Tian Y (2020). A novel method of calibrating micro-scale parameters of PFC model and experimental validation. Appl. Sci..

[36] Goldenberg C, Goldhirsch I (2008). Effects of friction and disorder on the quasistatic response of granular solids to a localized force. Phys. Rev. E..

[37] Jiang N (2023). Experimental study on mechanical properties of single fracture-hole red sandstone. Front. Earth Sci..

[38] Pan H, Jiang N, Gao Z, Liang X, Yin D (2022). Simulation study on the mechanical properties and failure characteristics of rocks with double holes and fractures. Geomech. Eng..

[39] Yao D, Jiang N, Wang X, Jia X, Lv K (2022). Mechanical behaviour and failure characteristics of rocks with composite defects of different angle fissures around hole. Bull. Eng. Geol. Environ..

[40] Xu Z (2008). Theory of Elasticity.

